# Diagnosis of miliary nodules as lung adenocarcinoma by cryobiopsy: A case report

**DOI:** 10.1111/1759-7714.13946

**Published:** 2021-03-23

**Authors:** Momoko Morishita, Manabu Suzuki, Hiromu Watanabe, Chie Morita, Akane Ishida, Masao Hashimoto, Go Naka, Yuichiro Takeda, Masayuki Hojo, Haruhito Sugiyama

**Affiliations:** ^1^ Department of Respiratory Medicine National Center for Global Health and Medicine Tokyo Japan

**Keywords:** *EGFR* mutation, liquid biopsy, metastatic lung tumor, T790M, TBLC

## Abstract

A 62‐year‐old woman with rheumatoid arthritis and a history of receiving immunosuppressant therapy had a recurrence of lung adenocarcinoma with EGFR L858R mutation. Following 14 months of treatment with erlotinib, computed tomography (CT) findings revealed the presence of small diffuse nodules. Bronchoscopy was performed as metastasis was suspected; however, this was not detected on lung biopsy with forceps. Transbronchial lung cryobiopsy (TBLC) succeeded in detecting metastatic adenocarcinoma, and T790M and L858R gene mutations. Pathological examination revealed a cluster of tumor cells in the intralobular interstitial areas, which was consistent with the CT findings. This report provides important information regarding the role of TBLC in diagnosing metastatic cancer, such as diffuse small miliary nodules, and its genetic mutations.

## Graphical abstract

This case report shows that cryobiopsy enables the diagnosis of miliary nodules as adenocarcinoma and its genetic mutations, and is a less invasive procedure than surgical lung biopsy. Cryobiopsy may be used to investigate malignant tumors and their genetic mutation in miliary nodules which liquid biopsy is unable to detect.

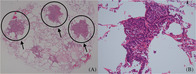

## INTRODUCTION

The demand for molecular profiling of tumors is steadily increasing for personalized cancer therapy. Rebiopsy is crucial for advanced treatment, especially in patients with gene mutations. For patients who have undergone treatment with a first or second‐generation epidermal growth factor receptor tyrosine kinase inhibitor (EGFR‐TKI), rebiopsy was positive for the T790M mutation in approximately 30%–68% of patients, thus, obtaining eligibility for treatment with third‐generation osimertinib.^1^, ^2^ Nowadays, T790M mutation can be detected by plasma testing. However, the detection rate of T790M in plasma with the cobas *EGFR* mutation test (Version 2) was only 9%–19.7%^1^, ^2^ therefore, obtaining tissue for testing is imperative.

In cases of recurrence, small nodules without the corresponding bronchi generally spread hematogenously. As diagnosis by transbronchial lung biopsy with forceps (TBLB) is difficult, some cases require surgical lung biopsy (SLB). However, in some cases, SLB should be avoided because of the invasiveness of surgery. Transbronchial lung cryobiopsy (TBLC) has recently replaced SLB for cases of interstitial lung disease because it provides larger and better‐preserved lung specimens than a forceps biopsy.^3^ Some investigators have reported that TBLC compared to TBLB, has contributed to a larger proportion of definite histomorphological diagnoses and a larger amount of DNA extracted from samples.^4^ Therefore, TBLC can be used for diagnosing lung cancer.

Some studies reported the peripheral mass diagnosis with TBLC,^5^ but no study to date has reported the diagnosis of smaller miliary nodules. This report describes a case where TBLC was used to achieve a successful diagnosis of diffuse small nodules, such as metastatic tumor and its gene mutation.

## CASE REPORT

A 62‐year‐old woman was referred to our department with a mass visible on chest radiograph. She had been receiving immunosuppressant therapy for rheumatoid arthritis over a 20‐year period and was a former smoker with a five pack‐year history. Computed tomography (CT) revealed a 31 mm mass in the left upper lobe. Bronchoscopic examination revealed lung adenocarcinoma, initially diagnosed as stage IIIA cT2aN2M0, after concurrent chemoradiotherapy as an induction treatment, followed by left upper lobectomy.

However, she reported back pain at two months postoperatively. Bone scintigraphy revealed hot spots on thoracic vertebrae T9 and 12, diagnosed as postoperative recurrence. Gene examination revealed exon 21 L858R mutation of *EGFR*, and erlotinib treatment was initiated.

**FIGURE 1:**
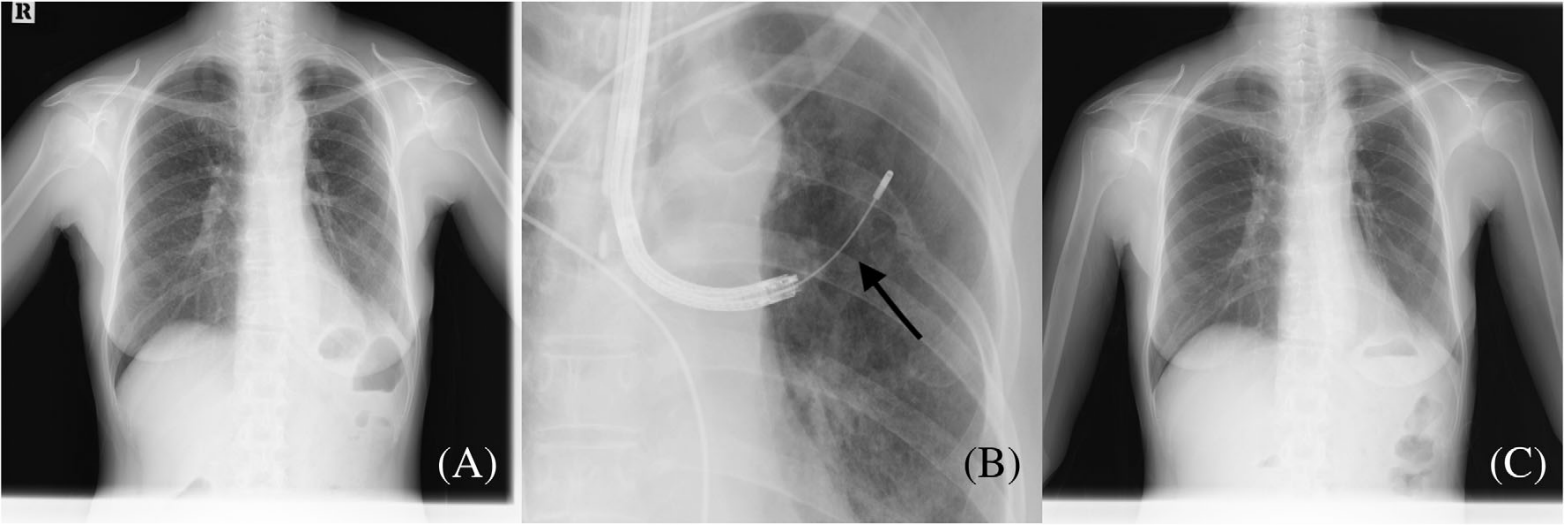
(a) Chest radiograph showed the presence of miliary nodules throughout both lungs. It was later diagnosed as metastatic tumors with L858R and T790M mutations. (b) Transbronchial lung cryobiopsy was performed in the left S6 area. (c) After treatment with osimertinib, the miliary nodules disappeared

**FIGURE 2:**
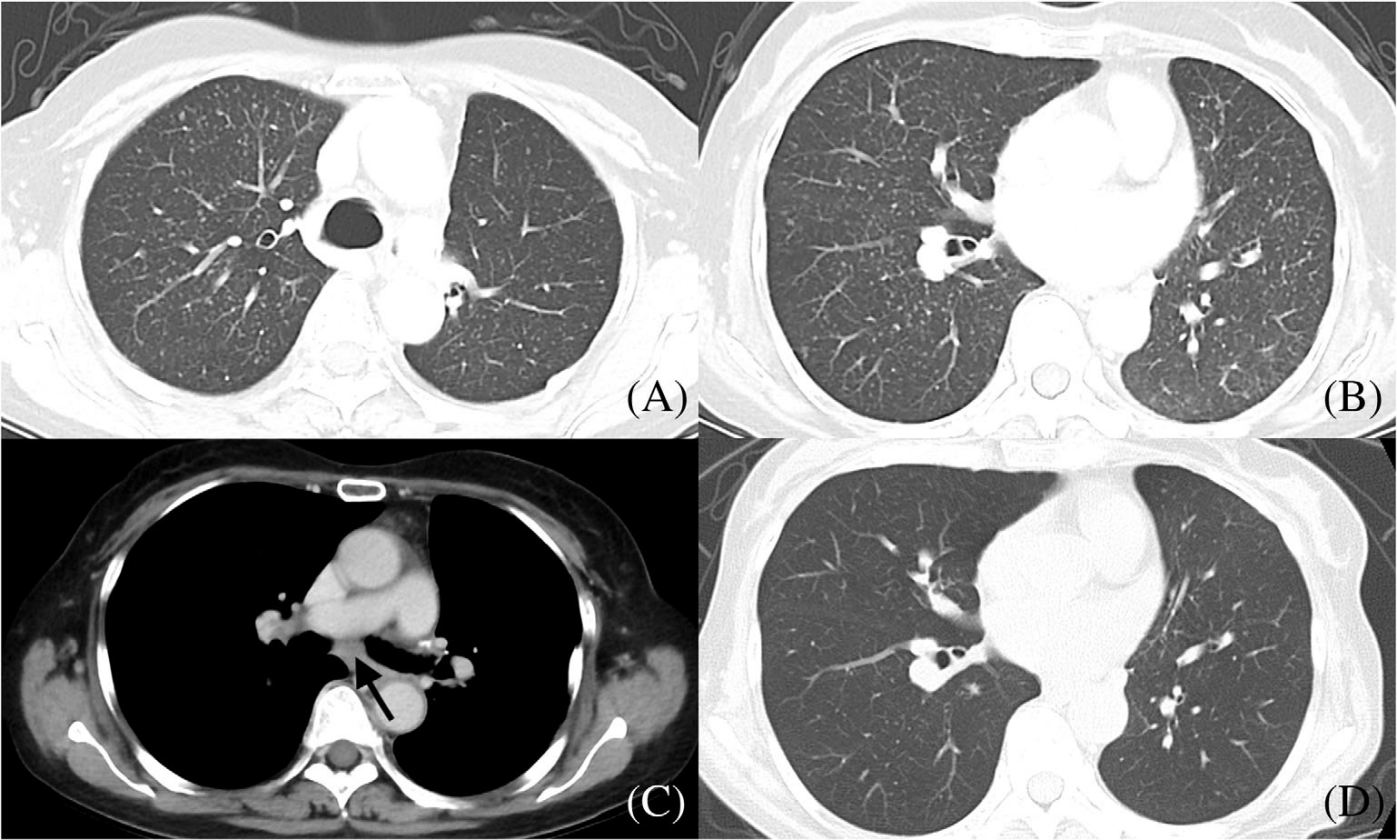
Thoracic computed tomography (CT) scan finding of the patient with miliary nodules. (a, b) CT showed the presence of miliary nodules. We performed transbronchial lung cryobiopsy (TBLC) and transbronchial lung biopsy with forceps (TBLB) to these areas and TBLC detected malignant tumors. (c) CT revealed swollen mediastinal lymph nodes. Transbronchial needle aspiration (TBNA) of the lymph node was performed but malignant cells were not detected. (d) CT did not reveal any miliary nodules after treatment with osimertinib

After 14 months, bone scintigraphy revealed other hot spots, and CT showed miliary nodules throughout both lungs with mediastinal lymph node enlargement (Figures 1(a) and 2(a)–(c)). Considering immunosuppressant therapy, tuberculosis was suspected. However, sputum culture and Interferon‐gamma release assays were negative. Therefore, the miliary nodules were considered as metastasis of lung adenocarcinoma, and liquid biopsy was conducted, which detected L858R but not T790M mutation. Two specimens from swollen mediastinal lymph nodes by EBUS‐guided transbronchial needle aspiration, six specimens from the left S10 by TBLB, and two specimens from the left S6 by TBLC (Figure 1(b)) were rebiopsied bronchoscopically for genomic precision medicine.

**FIGURE 3:**
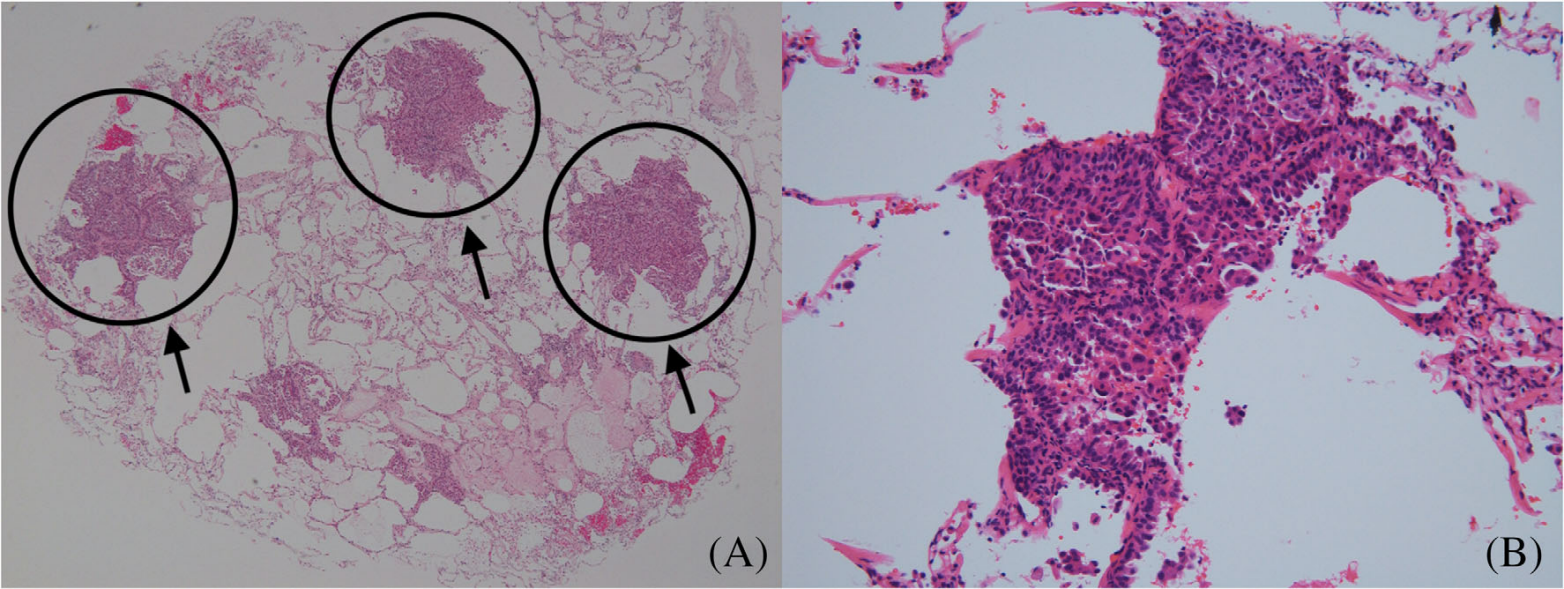
Pathological findings. Pathological examination of the transbronchial lung cryobiopsy specimen stained with hematoxylin and eosin. (a) Scattered masses of malignant cells (40× magnification), showing tumor cell clusters distributed in the intralobular interstitial areas without the corresponding bronchi. This might indicate that only transbronchial lung cryobiopsy (TBLC) could detect malignant cells and transbronchial lung biopsy with forceps (TBLB) could not. (b) At 400× magnification, atypical cells with anisonucleosis were observed in a micropapillary pattern consistent with adenocarcinoma

Only TBLC specimens detected malignant cells in which genomic examination revealed T790M (Figure 3(a), (b)). Figure 3(a) shows tumor cell clusters distributed in intralobular interstitial areas without the corresponding bronchi. Lung miliary nodules were not observed on the chest radiograph findings at one month after starting osimertinib use (Figures 1(c) and 2(d)).

## DISCUSSION

Over the last decade, genomic precision medicine has become inevitable among modern therapeutic strategies for lung cancer. In cases of *EGFR*‐mutated lung cancer, the detection of T790M is essential for deciding the treatment following initial EGFR‐TKI treatment failure.^1^ TBLC is a better diagnostic tool than TBLB for cases of interstitial lung disease and malignant tumors, as it provides larger and better‐preserved lung specimens.^3^

In our patient, after initial erlotinib treatment failure, CT scan showed multiple enlarged mediastinal lymph nodes and miliary nodules randomly distributed throughout the lung. As liquid biopsy failed to detect the T790M mutation, tissue rebiopsy was required before commencing osimertinib.^1^

Most pulmonary metastatic lesions occur hematogenously and are sometimes located only in interstitial lesions. In our case, pathological examination of the TBLC specimen showed a cluster of tumor cells distributed in intralobular interstitial areas, suggesting hematogenous metastasis. Rebiopsy using TBLC could detect malignant tumors and its genetic mutations, L858R and T790M. In some patients with lung cancer, miliary nodules are randomly distributed throughout their lungs, without solid nodules.^6^ These radiological findings were associated with a higher rate of *EGFR* mutations.^7^

This case report demonstrated that TBLC may be used to investigate malignant tumors and their gene mutations in miliary nodules randomly distributed throughout the lungs. A bronchoscopic approach could be difficult in rebiopsy cases as new lesions need to be rebiopsied more frequently than primary lesions, and new lesions are sometimes caused by hematogenous metastases via microvessels. Thus, TBLB was avoided because of the low diagnostic rates. The use of cryoprobes may allow the performance of bronchoscopic biopsy in the deterioration site and, simultaneously, allow the gene profile to be known.

Moreover, TBLC could bring some complications, such as pneumothorax and bleeding, but the rate of severe bleeding and adverse event occurrence is approximately 4%.^5^ According to Cooley et al., the adverse event rates are as follows: pneumothorax, 11%; persistent air leak, 1.3%; moderate–severe bleeding, 3.8%; ICU transfer within 48 h, 3.1%; and all cause 30‐day mortality, 1.9%. No deaths were attributed to the procedure.^8^ Furthermore, to prevent major bleeding, we used an endobronchial balloon catheter for bronchial blockade, which was reported to prevent blood from entering the central airways in cases of significant bleeding.^9^ Moreover, in our case, there were no major adverse events that could have resulted in prolonged hospital stay.

Conclusively, CT and pathological findings of the miliary nodules indicated difficulty in diagnosis with TBLB forceps, owing to the lack of bronchial involvement and the small size of the lesions at a distance from the bronchus. However, TBLC may enable the diagnosis of these pulmonary lesions and the detection of gene mutations without causing any severe complications. Thus, TBLC may be useful for the diagnosis of metastatic tumors with randomly distributed miliary nodules or lymphangitis carcinoma and, if diagnosed, its gene mutations.

## CONFLICT OF INTEREST

The funding source had no role in writing this report. The authors declare that they have no conflicts of interest.
